# Supplementary material for: Two Polysaccharides from Liupao Tea Exert Beneficial Effects in Simulated Digestion and Fermentation Model In Vitro
Source: Foods. 2022 Sep 21;11(19):2958. doi: 10.3390/foods11192958 (PMC9564304; doi:10.3390/foods11192958)
Supplement: Supplementary file 1 [file foods-11-02958-s001.zip › foods-1901594-supplementary.pdf]

foods

MDPI

Supplementary Materials

Two Polysaccharides from Liupao Tea Exert Beneficial Effects in Simulated Digestion and Fermentation Model In Vitro

Supplementary Figures:

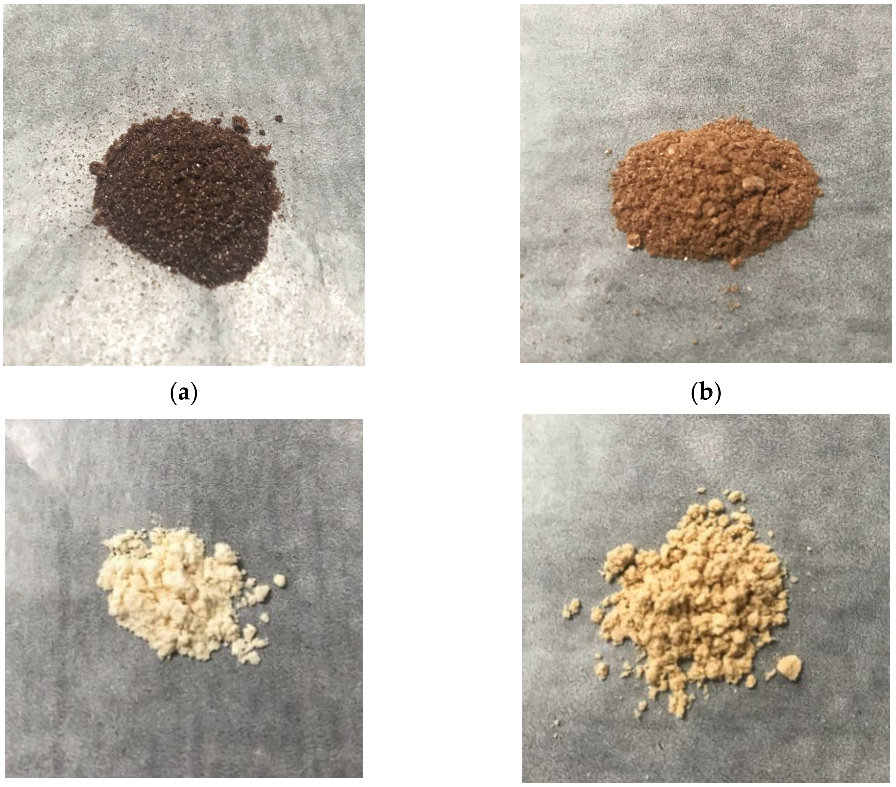

(**a**) (**b**) (**c**) (**d**)

Figure S1. The morphologies of crude TPS (**a**), refined TPS (**b**), TPS2 (**c**) and TPS5 (**d**).

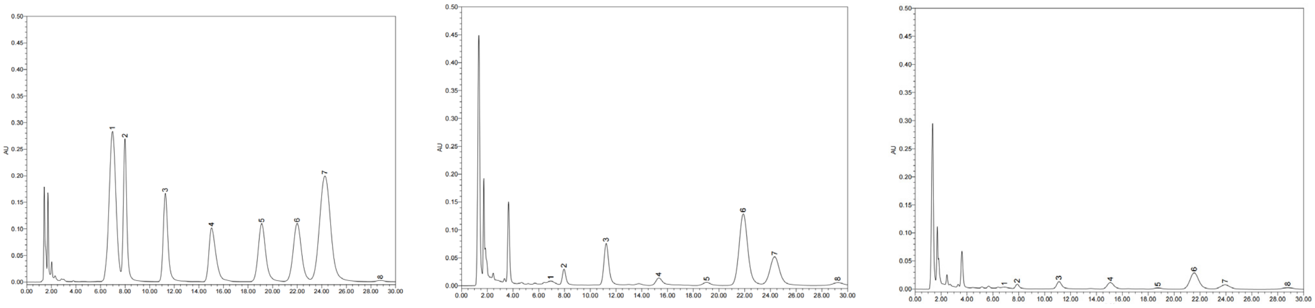

(**a**) (**b**) (**c**)

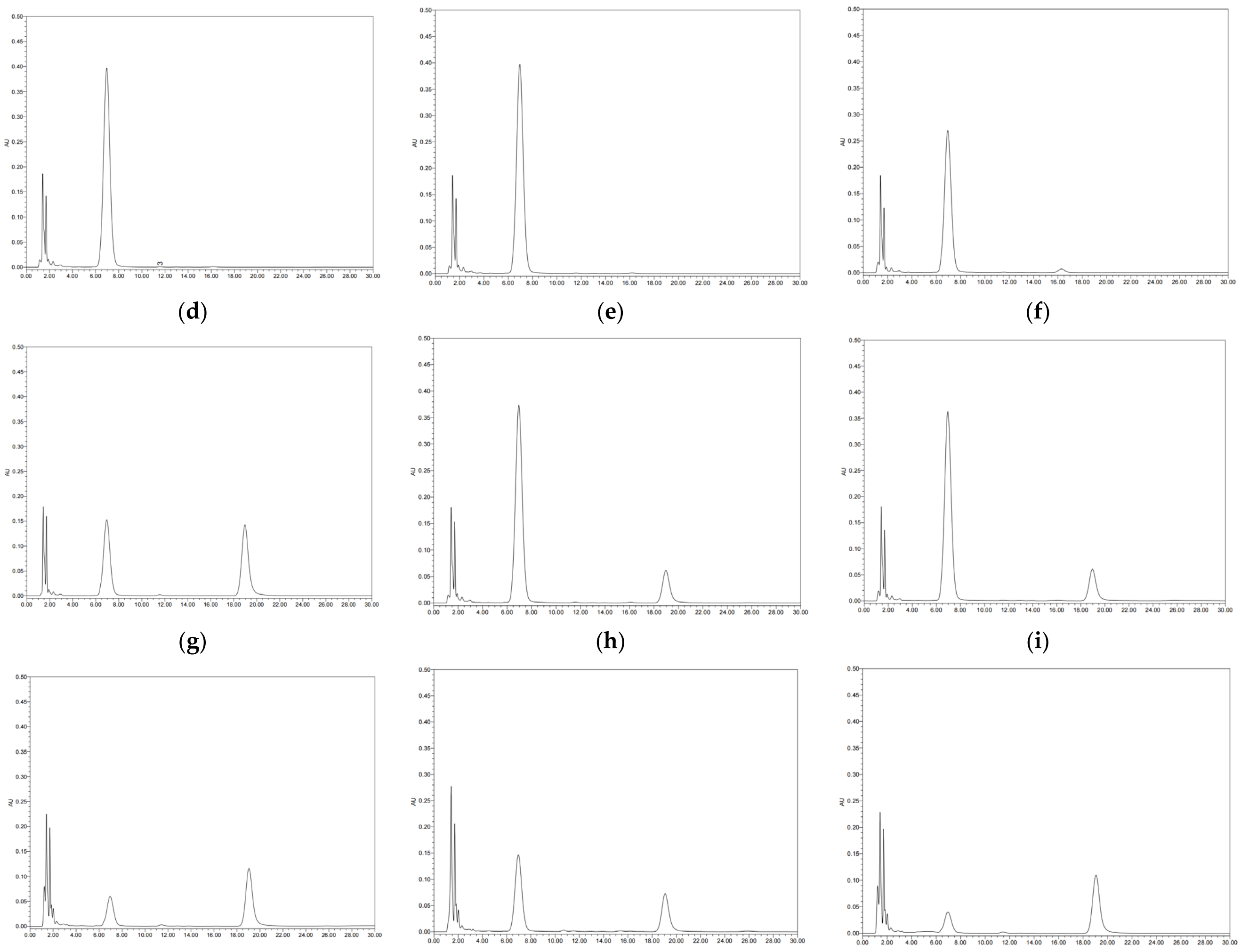

(**d**) (**e**) (**f**)

(**g**) (**h**) (**i**)

(**j**) (**k**) (**l**)

Figure S2. The HPLC chromatogram of monosaccharide composition of standard monosaccharides (**a**), TPS2 (**b**) and TPS5 (**c**); The HPLC chromatogram of free monosaccharide determination of the simulated salivary juice (**d**) and it digested with TPS2 (**e**) and TPS5 (**f**); The HPLC chromatogram of free monosaccharide determination of the simulated gastric juice (**g**) and it digested with TPS2 (**h**) and TPS5 (**i**); The HPLC chromatogram of free monosaccharide determination of the small intestinal juice (**j**) and it digested with TPS2 (**k**) and TPS5 (**l**). 1-PMP; 2-Man; 3-Rha; 4-GlcA; 5-Glc; 6-Gal; 7-Ara; 8-unknown.

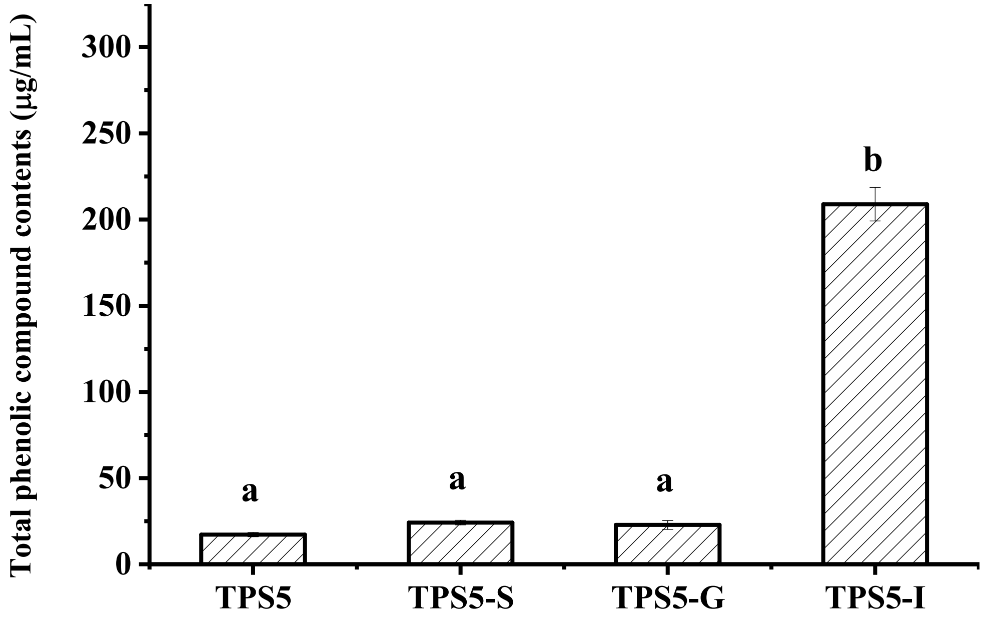

Figure S3. Changes of polyphenol content in TPS5 during simulated digestion. TPS5-S indicates TPS5 was digested by salivary; TPS5-G indicates TPS5 was digested by gastric juice and TPS-I indicates TPS5 was digested by intestinal juice. Different letters indicate significant difference ($p < 0.05$).

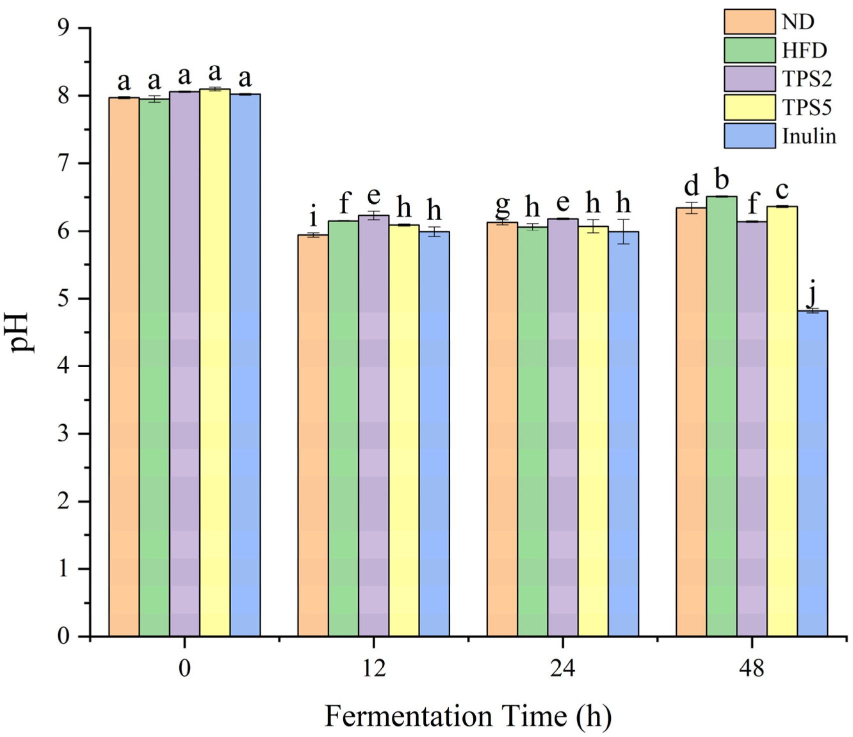

Figure S4. Changes in pH during *in vitro* fermentation. Different letters indicate significant difference ($p < 0.05$).

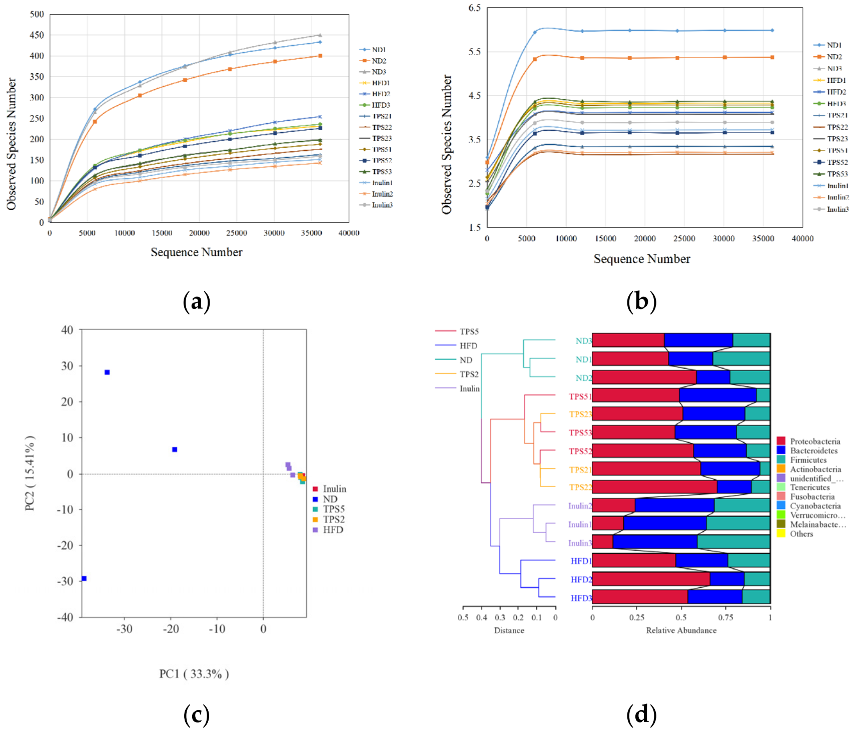

Figure S5. Alpha diversity analysis of samples rarefaction analysis (**a**) and Shannon curves (**b**). Principal coordinate analysis on Bray–Curtis distance (**c**) and clustering tree based on Bray–Curtis distance (**d**).

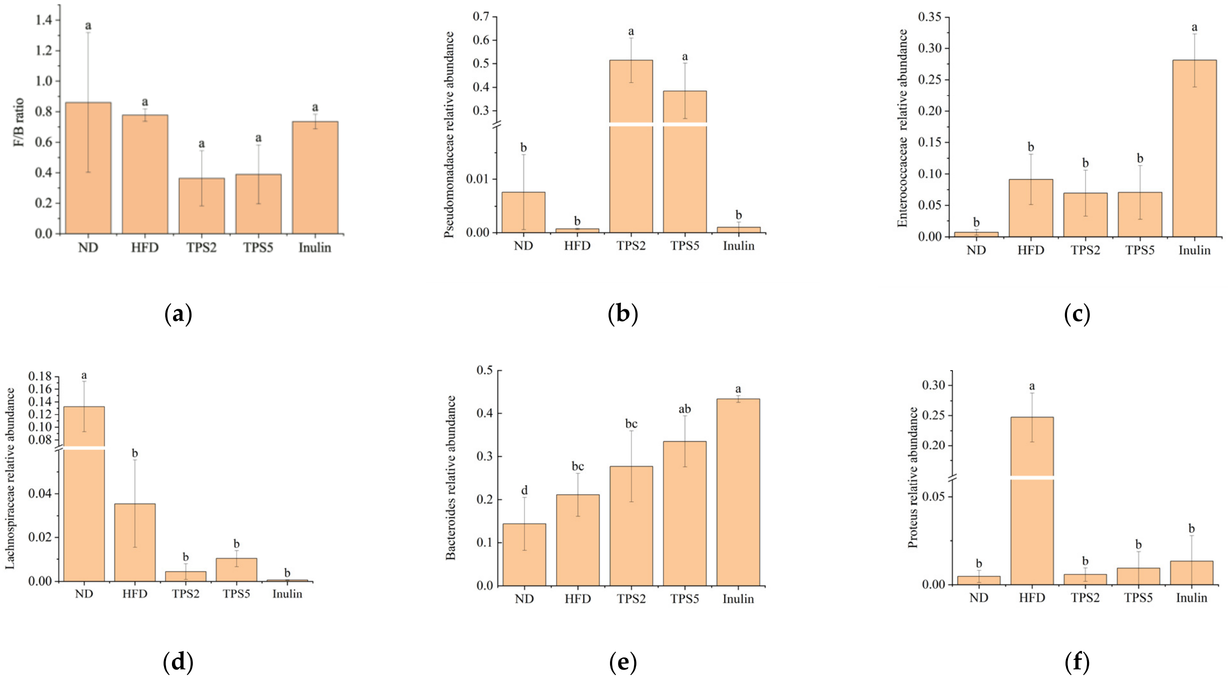

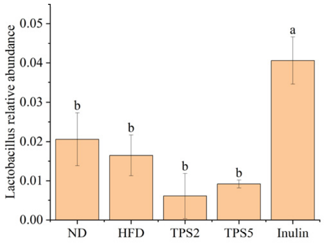

(**g**)

Figure S6. The F/B ratio (**a**) and the relative abundance of *Pseudomonadaceae* (**b**), *Enterococcaceae* (**c**), *Lachnospiraceae* (**d**), *Bacteroides* (**e**), *Proteus* (**f**), *Lactobacillus* (**g**). Different letters indicate significant difference ($p < 0.05$).

Supplementary Tables:

Table S1. Composition of normal diet and high fat diet.

Ingredient	Normal Diet a	High Fat Diet b
Corn	350	350
Wheat bran	150	150
Bean pulp	150	150
Soybean oil	100	100
Fish meal	80	80
Yeast	20	20
Mineral mixture	35	35
Vitamin mixture	10	10
L-cystine	3	3
Sucrose	0	100
Lard	0	100
Cholesterol	0	20
Choline chloride	0	2

Normal diet was purchased from the Beijing keaoxieli Feed Co., Ltd. The formulation of high fat feed consists of basal feed 68.6%, lard oil 10.0%, sucrose 10.0%, cholesterol 2%, and bile salt 0.2%.

Table S2. Serum lever of rats after 14 days animal model establishment.

Group (mmol/L)	ND-Iunduced Rats(n = 3)	HFD-Iunduced Rats(n = 12)
total cholesterol (TC)	1.27 ± 0.18	1.33 ± 0.21**
Triglyceride (TG)	0.33 ± 0.23	0.58 ± 0.12**
low density lipoprotein cholesterol (LDL-C)	0.51 ± 0.04	1.58 ± 0.19**

** indicates that there is a significant difference ($p < 0.01$).

Table S3. Statistics of sequences after treatment.

Sample	Raw_Reads	Clean_Reads	Base(nt)	AvgLen(nt)	Q20	GC%	Effective%
ND1	85077	80859	32930222	407	78.08	52.64	95.04
ND2	77414	74398	30157809	405	78.68	52.8	96.1
ND3	82554	80150	33254373	414	78.2	52	97.09
HFD1	79956	77260	32194701	416	77.38	51.67	96.63
HFD2	85763	80080	33170952	414	83.21	51.83	93.37
HFD3	87820	80143	33377425	416	82.55	51.23	91.26

TPS21	83427	80092	33217554	414	81.76	49.47	96
TPS22	82158	79838	32982138	413	82.74	50.52	97.18
TPS23	82223	80020	33164925	414	82.21	49.73	97.32
TPS51	84544	80106	32977192	411	80.29	50.05	94.75
TPS52	87126	80092	33315673	415	83.35	49.7	91.93
TPS53	82142	80079	33220931	414	82.31	49.73	97.49
Inulin1	88079	79903	33550921	419	79.92	50.02	90.72
Inulin2	86215	80174	33311608	415	85.01	50.55	92.99
Inulin3	81960	80088	33616300	419	79.78	49.8	97.72
